# Aorta unveiled: the crucial role of imaging in diagnosing and managing aortic disease—a review

**DOI:** 10.1093/ehjimp/qyaf108

**Published:** 2025-08-13

**Authors:** Jean-Baptiste Ricco, Aurélien Hostalrich, Xavier Chaufour

**Affiliations:** Department of Vascular Surgery, University Hospital of Rangueil, 1 avenue Jean Poulhes, Cedex 09 Toulouse 31059, France; University of Poitiers, Medical School, ABS-LAB, 2, rue de la Miletrie, 86021 Poitiers, France; Department of Vascular Surgery, University Hospital of Rangueil, 1 avenue Jean Poulhes, Cedex 09 Toulouse 31059, France; Haute-Garonne, Université de Toulouse, Inserm UMR 1297, UT3, Toulouse, France; Department of Vascular Surgery, University Hospital of Rangueil, 1 avenue Jean Poulhes, Cedex 09 Toulouse 31059, France; Haute-Garonne, Université de Toulouse, Inserm UMR 1297, UT3, Toulouse, France

**Keywords:** aortic imaging, computed tomography angiography, magnetic resonance imaging, hybrid imaging, artificial intelligence, contrast-enhanced ultrasonography

## Abstract

Aortic diseases, including aneurysmal and occlusive pathologies of the thoracic and abdominal aorta, represent a significant source of cardiovascular morbidity and mortality. This narrative review explores the role of modern and emerging imaging modalities in the management of aortic disease and highlights the pivotal roles of computed tomography angiography (CTA), magnetic resonance imaging (MRI), and contrast-enhanced ultrasound (CEUS).

CTA remains the cornerstone for evaluating aneurysms, dissections, and traumatic injuries, offering high spatial resolution, rapid acquisition, and detailed anatomical assessment. MRI, particularly with advanced sequences such as 4D flow, provides comprehensive multiparametric evaluation without radiation exposure, making it ideal for younger patients and those requiring repeat imaging. Positron emission tomography (PET), especially when integrated with CTA or MRI, enables metabolic characterization of inflammation and infection in aortic walls. Ultrasound, particularly CEUS, remains indispensable in abdominal aortic aneurysm (AAA) screening and post-endovascular aortic aneurysm repair (EVAR) surveillance, especially in patients with renal impairment.

Emerging technologies, including hybrid imaging, radiomics, and artificial intelligence (AI) are reshaping the landscape of aortic diagnostics. These innovations enhance detection of subtle imaging features, automate measurements, and may enable prediction of disease progression or complications.

## Graphical Abstract

**Graphical Abstract:**
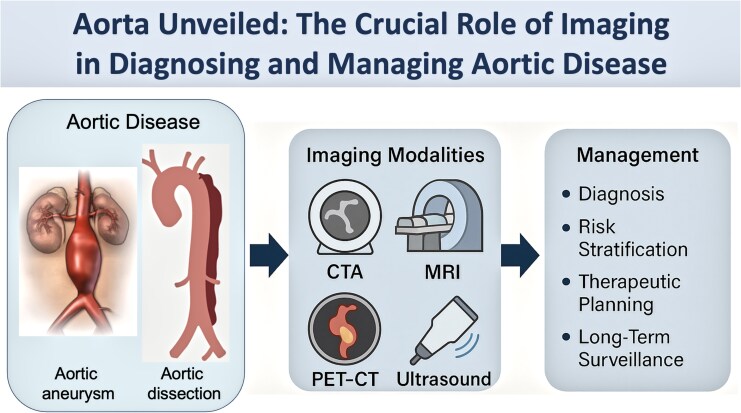

## Introduction

Aortic diseases are associated with significant morbidity and mortality. Conditions such as thoracic and abdominal aortic aneurysms (AAAs), aortic dissections, and chronic occlusive disease necessitate timely diagnosis and tailored management.^1,2^ Imaging plays a central role in every phase of care, from initial detection in asymptomatic patients to long-term surveillance. The continuous evolution of imaging technologies has enhanced diagnostic accuracy and treatment outcomes. This narrative review explores the role of modern and emerging imaging modalities in the management of aortic disease.

## Computed tomography angiography

**Figure 1:**
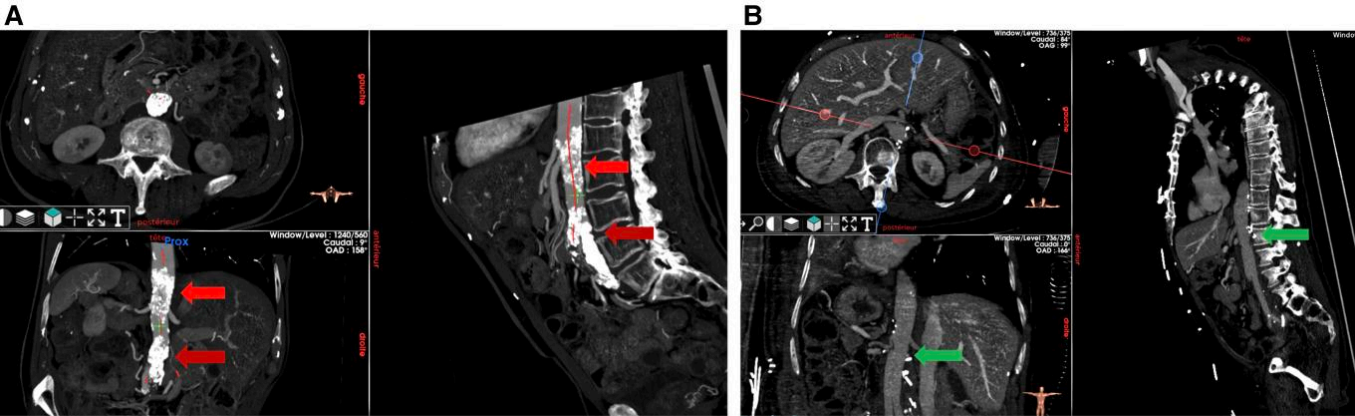
(*A*) Preoperative multiplanar reconstruction scan showing a coral reef of the visceral aorta associated with infrarenal aortic occlusion. The red arrows show the coral reef at the level of the coeliac aorta and at the level of the infrarenal aorta. (*B*) Postoperative CTA of the same patient showing patency of the visceral aorta, renal and digestive arteries after endarterectomy, and aorto-bifemoral bypass. Green arrows show a patent coeliac and infrarenal aorta without residual calcified lesions.

Computed tomography angiography (CTA) has emerged as the cornerstone imaging modality for the comprehensive evaluation of aortic diseases. It offers unparalleled anatomical detail crucial for accurate diagnosis, precise treatment planning, and effective post-intervention surveillance (*Figure 1*). Its widespread availability, rapid acquisition times, and high spatial resolution solidify its role as the preferred imaging technique for a broad spectrum of aortic pathologies.^1,3–5^

### Considerations in imaging with CTA

#### Radiation exposure

Strategies to minimize radiation doses are continuously evolving. These include lower tube voltage (kVp), automated tube current modulation, and iterative reconstruction techniques.

#### Contrast-induced nephropathy (CIN)

The use of iodinated contrast media may be contraindicated in patients due to previous severe allergic reactions and in patients’ advanced chronic kidney disease.

### CTA for aortic aneurysm

**Figure 2:**
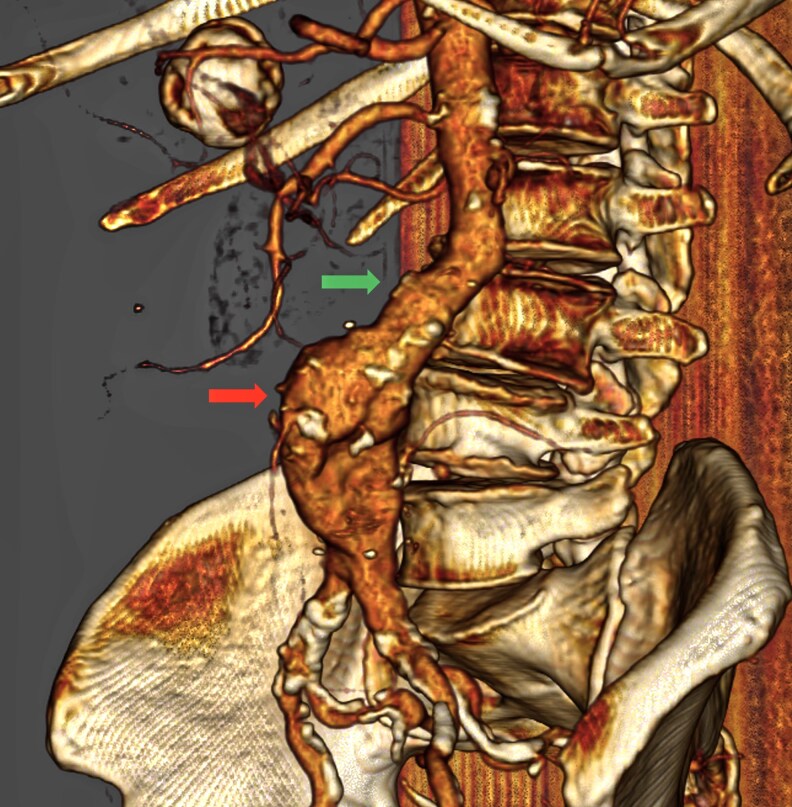
Volume rendering from a CTA showing a large aneurysm (6 cm in diameter) of the infrarenal aorta. The green arrow shows the proximal neck of the aneurysm, and the red arrow the aneurysm sac.

AAAs are defined as a localized increase in diameter of more than 50% compared with the normal adjacent segment, or an absolute diameter exceeding 3.0 cm for the abdominal aorta (*Figure 2*). Early detection and meticulous monitoring are paramount due to the inherent risk of rupture.^2^ Two recent European national screening programmes report an AAA prevalence of <1%. In contrast, a screening programme in the USA, only offering screening to current and ex-smokers, reported prevalence of >5%.^1^

- *Specificities and diagnostic capabilities*

*Precise measurement:* CTA allows for accurate determination of aneurysm dimensions (*Table 1*), including the maximum transverse diameter perpendicular to the central flow axis. These measurements are critical for assessing rupture risk and determining the optimal timing and type of intervention. For instance, current guidelines often consider intervention for AAAs when the diameter equals or exceeds 5.5 cm in men and 5.0 cm in women, with lower thresholds for patients with connective tissue disorders.^4^ Furthermore, CTA offers the possibility to concomitantly assess coronary artery disease and also cardiac function with full-beat cardiac CT (4-slice multidetector CT) (MDCT). In addition, recent guidelines have suggested the use of CTA or magnetic resonance angiography (MRA) very early in patients with aortic dilatation to confirm the initial dimension and validate echo measurements.^1,2^ These recommendations lead to a word of caution. They can lead to performing numerous CTA or MRA, at a significant economic cost, in patients with the aorta, far from the limits that would require intervention. However, CTA and MRA confirm the exact diameter of the aorta, allowing for correlation with ultrasound for follow-up without having to repeat CTA or magnetic resonance imaging (MRI). Therefore, early CTA or MRI seems to be a logical option, especially combined with artificial intelligence (AI), which allows for an objective calculation of aortic volume.

- *Morphological assessment*

**Table 1:** CTA

Specificities	Details
Spatial resolution	Excellent (submillimetric)
Speed	Rapid acquisition suitable for emergencies
3D reconstruction	Readily available, essential for planning
Radiation	Yes, significant exposure
Contrast agent	Iodinated (risk of nephrotoxicity)
Best use	Acute aortic syndromes, trauma, preoperative planning, aortic aneurysm

CTA offers a detailed characterization of AAA morphology. It also identifies the presence and extent of mural thrombus, calcification, and the relationship between aneurysm and critical branch vessels, such as the renal and mesenteric arteries, which is vital for open aortic surgical repair (OASR) or endovascular planning.

- *Identification of impending rupture or complications*

**Figure 3:**
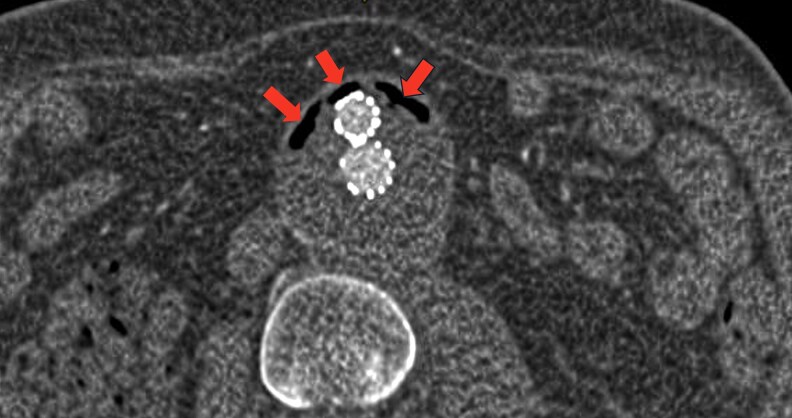
Cross-sectional CTA showing endovascular exclusion of an infrarenal aortic aneurysm with the presence of air within the aneurysm sac indicating an endoprosthesis infection (red arrows).

CTA can detect signs indicative of acute or impending rupture, which are surgical emergencies. These signs include:

- Retroperitoneal or mediastinal haematoma
- Peri-aortic stranding: soft tissue changes around the aorta indicative of inflammation or contained leak
- High-attenuation crescent sign: a hyperdense crescent within the mural thrombus on non-contrast CT, suggesting an acute or impending rupture
- Active contrast extravasation: direct visualization of contrast material leaking outside the aortic lumen, confirming active bleeding

- *Pre-procedural planning for endovascular aortic aneurysm repair and for OASR*
  For patients undergoing post-endovascular aortic aneurysm repair (EVAR), CTA is required for preoperative planning:
  ∘ *Landing zone evaluation:* it precisely assesses the proximal and distal aortic landing zones—the healthy segments of the aorta where the stent graft will be deployed and sealed. This includes measuring diameters, lengths, and angulation to ensure a secure and durable seal.^6^
  ∘ *Branch vessel mapping:* detailed mapping of the origins of aortic branch vessels (e.g. renal, superior mesenteric, and coeliac arteries) is crucial to plan for standard, fenestrated, or branched endografts, ensuring perfusion to vital organs is maintained.
  ∘ *Access vessel assessment:* CTA evaluates the iliac and femoral arteries for calcification, tortuosity, or stenosis, which can impact the feasibility and safety of endograft delivery.
- *Post-procedural surveillance for EVAR*
  CTA is fundamental for long-term surveillance following EVAR to detect complications, primarily endoleaks, which can lead to continued aneurysm expansion and rupture but also endograft infection (*Figure 3*).
  ∘ *Protocol:* triple-phase CTA (non-contrast, arterial, and delayed phases) is often employed to maximize sensitivity for endoleak detection. Non-contrast images help differentiate enhancing endoleaks from pre-existing calcifications or thrombus.^7^
  ∘ *Guidelines:* Lifelong surveillance is recommended after EVAR, typically with an initial CTA within 30 days post-procedure, followed by regular imaging intervals depending on the initial findings and aneurysm stability.^8^

### CTA for aortic dissection

Aortic dissection is an acute condition characterized by an intimal tear that allows blood to dissect into the medial layer of the aortic wall, creating a true lumen and a false lumen. Rapid and accurate diagnosis is critical due to the high mortality associated with delayed intervention.

- *Imaging protocol for aortic dissection*

*Comprehensive coverage:* imaging should ideally extend from the neck to assess supra-aortic vessels through the entire thoracic and abdominal aorta down to the femoral arteries to capture the full extent of the dissection and any associated malperfusion.

*ECG gating:* ECG-gated CTA, particularly for the ascending aorta and aortic arch, is highly recommended. Synchronized CTA has better diagnostic performance for ascending and aortic arch dissection than non-synchronized CTA. However, the superiority of synchronized CTA is not demonstrated for the descending thoracic aorta or for the abdominal aorta.^9,10^

*Contrast enhancement:* intravenous iodinated contrast is crucial for visualizing the true and false lumens and assessing their dynamic opacification. A non-contrast phase is also valuable, especially for detecting intramural haematomas and distinguishing thrombus from acute haemorrhage.

**Figure 4:**
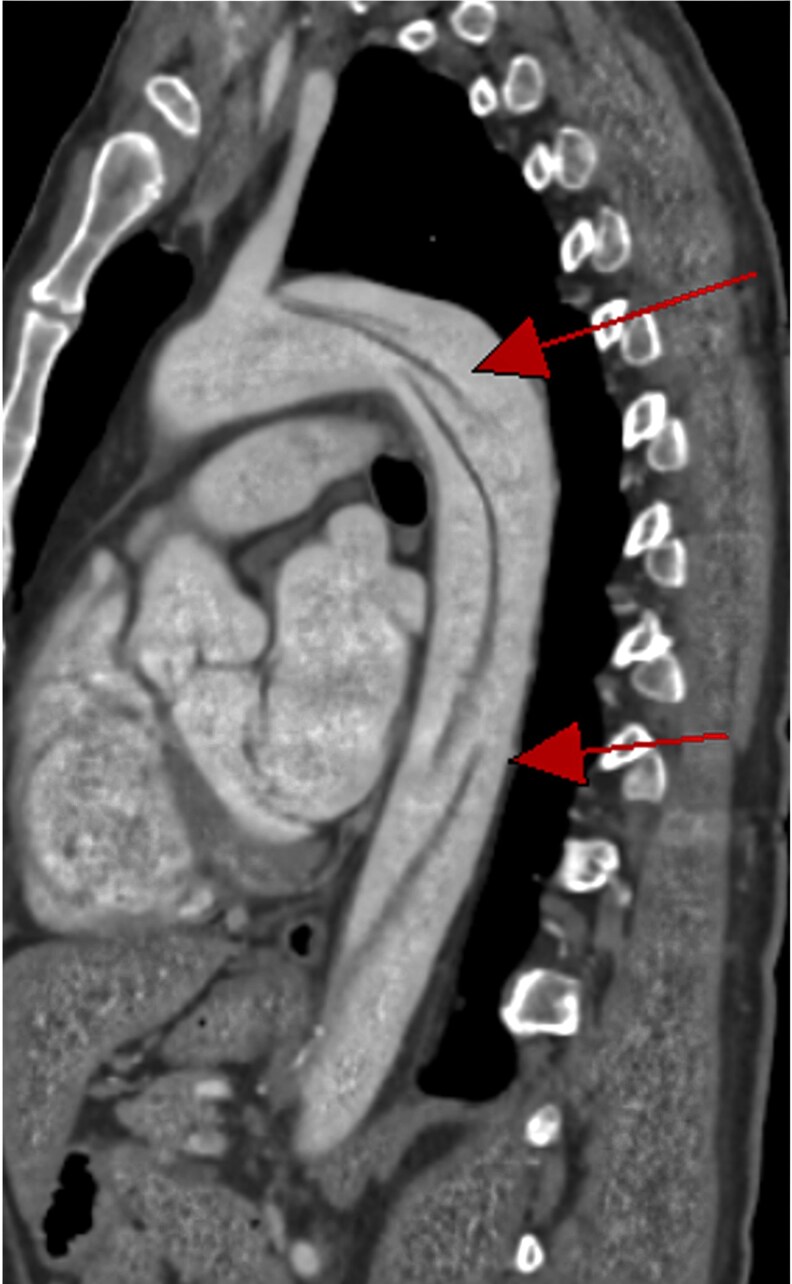
CTA of the thoracic aorta (sagittal section) showing a TBAD with two entry tears below the left subclavian artery (red arrows).

- *Diagnostic capabilities*
  CTA is the preferred imaging modality for suspected acute aortic syndrome due to its speed, widespread availability, and high diagnostic accuracy, with reported sensitivity and specificity approaching 100%.^1^ Key CTA findings include the following:
  ∘ *Intramural haematoma (IMH):* CTA readily identifies IMH as a crescent-shaped, high-attenuation thickening of the aortic wall on non-contrast images, without an intimal flap. IMH is considered a variant of acute aortic syndrome and may progress to classical dissection or rupture.^11,12^
  ∘ *Intimal flap:* the hallmark of dissection is the visualization of a thin, linear intimal flap separating the true lumen from the false lumen, often larger, with delayed or no opacification if thrombosed.^13^
  ∘ *True vs. false lumen differentiation:* distinguishing between the true and false lumens is crucial. The true lumen typically enhances earlier and more homogeneously, often retains calcifications from the original aortic wall, and gives rise to major branch vessels. The false lumen often shows delayed enhancement and may be larger and partially or completely thrombosed, and the left renal artery frequently originates from it.^14^
  ∘ *Extent and classification:* CTA accurately depicts the full craniocaudal extent of the dissection, from the aortic root to the iliac arteries. This allows for precise classification, e.g. Stanford Type A involving the ascending aorta; and Stanford Type B (*Figure 4*) limited to the aorta distal to the left subclavian artery.^2^
  ∘ *Evolution of the aortic diameter:* it is an important criterion for thoracic aortic aneurysm endovascular repair (TEVAR) in uncomplicated Type B aortic dissections (TBADs). TEVAR is indicated in the acute phase only in cases of complications: persistent pain, false lumen expansion, impending rupture, and organ ischaemia. However, certain morphological criteria predictive of adverse outcome (CTA) may indicate early TEVAR: total aortic diameter > 40 mm, large false lumen (>22 mm), and large proximal inlet (>10 mm). These factors must be interpreted on a case-by-case basis, as a part of a multidisciplinary discussion.
  ∘ *Branch vessel involvement:* CTA is essential for identifying involvement and potential compromise perfusion of major aortic branch vessels (e.g. coronary arteries, brachiocephalic arteries, mesenteric arteries, and renal arteries). Ischaemia in these territories can significantly impact patient morbidity and mortality.^2^
  ∘ CTA is indispensable for the serial surveillance of known aortic dissections, particularly stable chronic Type B dissections managed medically. Follow-up imaging allows monitoring for changes in lumen size, false lumen thrombosis, and the development of new complications such as aneurysmal degeneration of the false lumen, which may need intervention.^15^
- *CTA for complications*
  ∘ CTA allows for the detection of critical complications in aortic dissection.
  ∘ Rupture: indicated by haemopericardium, haemothorax, or mediastinal haematoma.^16^
  ∘ Aortic insufficiency: although echocardiography is superior for assessing valve function, CTA may show features of significant aortic root dilatation.
  ∘ Visceral or limb ischaemia, due to malperfusion of aortic branch vessels.

### CTA in aortic trauma

**Figure 5:**
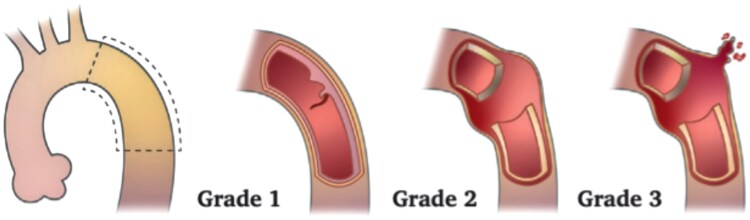
ESVS classification by CTA of traumatic thoracic aortic injury: ESVS Grade 1, injury confined to the intima or vessel wall with normal external wall contour; ESVS Grade 2, abnormal external wall contour or external wall disruption with contained haemorrhage (e.g. pseudoaneurysm); and ESVS Grade 3, complete wall transection with free rupture.

CTA is also central to trauma imaging. According to the 2025 European Society for Vascular Surgery (ESVS) Guidelines on the Management of Vascular Trauma,^17^ CTA including arterial and venous phases is the first-line imaging modality for suspected blunt thoracic aortic injury (BTAI). It allows for lesion grading (*Figure 5*), from intimal tear (Grade 1) to pseudoaneurysm or rupture (Grades 2 and 3), and guides timing and method of intervention (*Table 2*). Endovascular repair is generally preferred, with sizing recommendations including 20–30% oversizing to accommodate hypovolaemia-induced underestimation of aortic diameter in hypovolaemic patients.^17,18^

**Table 2:** Suggested management approach for BTAI based on grade of aortic injury on CTA

ESVS grade	SVS grade	Description	Management
1	I and II	Injury confined to the intima or vessel wall with normal wall contour	Non-operative: repeat CTA within 48–72 h to assess aortic lesion stability
2	III	Abnormal external wall contour or external wall disruption with contained haemorrhage (e.g. pseudoaneurysm)	Low-risk BTAI features: delayed (>24 h) TEVAR. Stabilization of other major traumatic injuries first
3	IV	Complete wall transection with free rupture	Emergency TEVAR

### Recent advancements in CTA for aortic imaging

Technological advancements continue to refine CTA’s capabilities in aortic imaging:

- *Faster acquisition:* Modern MDCT scanners with increased gantry rotation speed and detector rows allow for sub-second scanning of the entire aorta, minimizing motion artefacts and enabling imaging during a single breath hold, together with the use of smaller contrast volumes.^19^
- *Dual-energy CT (DECT):* DECT, by acquiring data at two different energy levels, offers advantages such as material decomposition (e.g. differentiating iodine from calcium), which is useful in evaluating endoleaks or thrombus, also reducing beam-hardening artefacts, and potentially lowering contrast agent volumes and radiation doses.^20,21^
- *AI and machine learning:* AI algorithms are increasingly being developed to automate aortic measurements, detect subtle imaging features of dissection or aneurysm on non-contrast CT, and predict disease progression or rupture risk. These tools hold promise for improving efficiency and diagnostic accuracy, especially in high-throughput settings.^22,23^
- *Image reconstruction techniques:* Advanced iterative reconstruction algorithms reduce image noise and allow for significant reductions in radiation dose while maintaining diagnostic image quality.

In conclusion, CTA remains the cornerstone of modern aortic imaging for both aneurysmal disease and dissection. Its continued evolution with faster acquisition, advanced post-processing, and integration of AI promises even greater precision and clinical utility in the diagnosis, management, and long-term surveillance of complex aortic pathologies.

## MRI for aortic diseases

MRI is a robust alternative to CTA, especially in younger patients or those requiring repeated imaging, due to its lack of ionizing radiation and superior soft tissue contrast (*Table 3*). It is distinctively positioned to provide multiparametric, dynamic, and tissue-specific information in the evaluation of the aorta.^24,25^ However, MRI cannot be used in patients with non-MRI–compatible devices, and gadolinium should not be used in patients with advance chronic kidney disease due to the risk of nephrogenic systemic fibrosis.

**Table 3:** MRI

Specificities	Details
Radiation	None (safe for repeated imaging)
Soft tissue contrast	High (visualizes vessel wall and flow)
Advanced Sequences	PC-MRI, 4D flow, black-blood imaging
Limitations	High cost, longer acquisition, contraindications (e.g. pacemakers)
Best use	Aortic dissection, connective tissue disorders

One of the key advantages of MRI in aortic imaging is its ability to perform phase-contrast MRI (PC-MRI), which quantifies blood flow velocity and direction through aortic segments. PC-MRI enables the evaluation of flow patterns, pulse wave velocity (PWV), wall shear stress, and turbulence, which are important parameters in assessing aortic stiffness and the risk of aneurysm progression.^26^

In chronic aortic dissections, MRI can accurately differentiate between true and false lumens, detect partial thrombosis, and assess branch vessel perfusion in patients with a TBAD which carries a prevalence between 2.9 and 4.0 per 100,000 person-years.^1,2^

**Figure 6:**
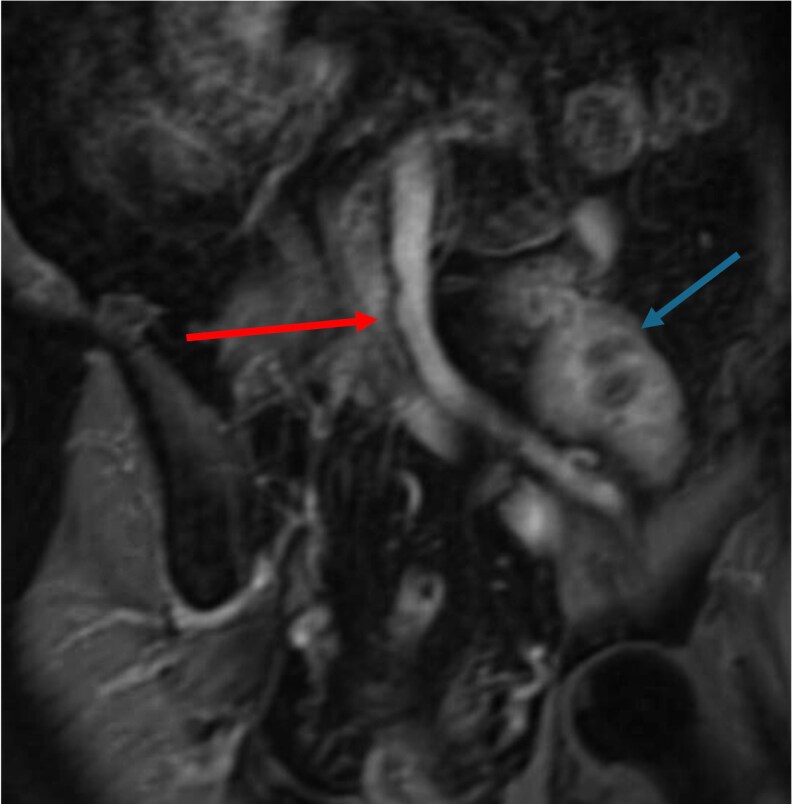
MRA showing an occluded right iliac artery (red arrow) in a patient with kidney transplant in the left iliac fossa (blue arrow).

Time-resolved contrast-enhanced MR angiography (TR-MRA) and non-contrast black-blood sequences are particularly valuable for identifying intimal flaps and visualizing flow within dissected segments. These techniques are crucial in patients with contraindications to contrast agents, such as those with renal insufficiency or recent kidney transplant (*Figure 6*). In patients with connective tissue disorders such as Marfan and Loeys–Dietz syndromes, MRI is the imaging modality of choice for long-term surveillance. It allows repeated whole-aorta assessment without radiation and facilitates evaluation of associated cardiac anomalies such as aortic valve insufficiency, mitral valve prolapse, or ventricular function.^27,28^

A major recent innovation is 4D flow MRI, which captures dynamic volumetric blood flow in three spatial dimensions over time. This technique provides detailed assessment of helical flow, vortices, and flow jets and enables visualization of abnormal flow patterns associated with conditions such as bicuspid aortic valve (BAV) and thoracic aortic diseases. Quantitative 4D flow parameters—such as flow displacement and eccentricity indexes—are emerging as biomarkers for predicting aortic dilation or dissection risk, particularly in asymptomatic patients.^29,30^

In addition, advanced T1 and T2 mapping techniques are being explored for tissue characterization, including detection of fibrosis, oedema, or inflammation in the aortic wall, which may play a role in aortitis, mycotic aneurysms, or chronic inflammation seen in vasculitis. Emerging techniques include ultrasmall superparamagnetic iron oxide-enhanced MRI (using ultrasmall superparamagnetic iron oxide particles) for detection of macrophage activity and vascular inflammation, offering a potential marker for aortic wall instability.

Finally, AI and machine learning approaches are now being integrated into MRI, particularly in aortic segmentation, flow quantification, and predictive modelling. AI-assisted analysis can reduce post-processing time for 4D flow data, enhance reproducibility, and support risk stratification for endoleak, rupture, or need for surgery.^31^

## Positron emission tomography scan and computed tomography and molecular imaging for aortic diseases

Positron emission tomography scan and computed tomography (PET–CT) integrates metabolic and anatomical imaging, offering unique insights into the inflammatory and infectious processes of the aortic wall (*Table 4*). Among the available radiotracers, ^18^F-fluorodeoxyglucose (FDG) is the most widely used. Increased FDG uptake has been correlated with active inflammation, extracellular matrix degradation, and cellular infiltration and is considered a surrogate marker for aneurysm instability and rupture risk, suggesting a possible role in improving risk stratification beyond size criteria.

**Table 4:** PET–CT

Specificities	Details
Radiation	Yes (significant cumulative dose)
Metabolic information	Detects inflammation and infection
Indications	Inflammatory aneurysms, vasculitis, graft infection
Limitations	Costly, less accessible
Best use	Research, selected clinical cases

The use of ^18^F-FDG PET–CT is particularly promising in patients presenting with an aortic inflammatory disease, such as Takayasu arteritis and giant cell arteritis, where conventional imaging may underestimate disease activity.^32,33^ It helps distinguish active inflammation from fibrotic or chronic disease stages, guiding immunosuppressive therapy and monitoring treatment response.^34,35^

Moreover, PET–CT plays a central role in detecting vascular graft infections, especially in late-onset prosthetic infections where conventional imaging is often inconclusive. The European Society of Vascular Surgery now acknowledges FDG PET/CT as a valuable diagnostic tool.^1,36^

Beyond FDG, new tracers are under investigation to improve specificity and provide more mechanistic insights. These include radiolabelled tracers targeting matrix metalloproteinases (MMPs), somatostatin receptors (e.g. ^68^Ga-dotatate), and macrophage activity (e.g. ^18^F-FMCH), though their clinical use remains investigational.

Despite its promise, PET–CT has several limitations: high cost, radiation burden, limited spatial resolution, and restricted availability. Nonetheless, it remains a powerful adjunct in selected cases and ground for translational research. Emerging PET–MRI hybrids offer further potential by combining high-resolution anatomical and soft tissue contrasts with molecular characterization, although clinical adoption is still limited.^37^

## Hybrid imaging and AI in aortic diseases

Hybrid imaging modalities, notably PET/CT and PET/MRI, combine anatomical precision with molecular and metabolic insights, providing a comprehensive view of complex vascular pathologies (*Table 5*).^38^ PET/MRI, though currently confined largely to research settings, offers several theoretical advantages, including superior soft tissue contrast, improved vascular wall delineation, and reduced radiation exposure. This modality allows for high-resolution inflammation mapping in diseases such as vasculitis or chronic aortic syndromes.^37,39^

**Table 5:** Hybrid imaging

Specificities	Details
Modalities	PET/CT, PET/MRI
Benefits	Combines anatomical and metabolic insights
Radiation	Reduced in PET/MRI
Use in aorta	Potential for complex or inflammatory aortopathies
Best use	Research, integrates inflammation with morphology assessment

The integration of AI into aortic imaging workflows represents a transformative shift (*Table 6*). AI-driven tools, especially those based on deep learning, have shown strong potential in automating critical imaging tasks, such as aortic diameter measurement, segmentation of aneurysmal sac, and identification of subtle morphological features, including ulcer-like projection and intramural haematoma. As shown by Fortuni *et al.*^40^ these tools increase reproducibility, reduce interobserver variability, and save time in large-volume imaging centres.

**Table 6:** AI imaging

Specificities	Details
Functions	Automated measurement, classification, prediction
Applications	Endoleak detection, aneurysm rupture risk
Techniques	Radiomics, deep learning, segmentation
Limitations	Need for large datasets validation
Future role	Integration into clinical workflow, decision support

Machine learning models incorporating radiomic features—quantitative texture data extracted from imaging modalities such as CTA or MRI—are being explored for risk stratification of aneurysmal growth and rupture. These features may reflect underlying biological phenomena such as microcalcification, wall stress, or mural inflammation not directly visible to the human eye.^22,23,41^ In the context of EVAR surveillance, AI-based approaches are being used to streamline interpretation of multiphase CTA, enhancing detection and classification of endoleaks. Novel convolutional neural networks (CNNs) can distinguish between Type I and Type II endoleaks with high accuracy, supporting more personalized follow-up strategies.^42,43^ AI also facilitates post-processing of advanced modalities such as 4D flow MRI, reducing manual workload and enabling real-time haemodynamic visualization or creating digital twins for predictive planning of endovascular aortic procedures.^44^

Although regulatory and implementation barriers remain, AI-assisted imaging in aortic disease is rapidly evolving, with several pilot studies and multicentre trials underway. Future directions include integration into clinical decision support systems and combination with genomic and clinical data for multimodal prediction.

## Ultrasound and contrast-enhanced ultrasound

Ultrasound remains a cornerstone in the diagnosis and surveillance of AAA.^45^ It is non-invasive, widely available, cost-effective, and free from ionizing radiation or nephrotoxic contrast agents. It allows for accurate aortic diameter measurement in both transverse and longitudinal planes and serves as the preferred modality in screening programmes, particularly in older men, as recommended by the ESVS guidelines.^1^

Ultrasound can also be used to also assess the aortic root, ascending aorta, and arch, both with transthoracic and with transoesophageal echocardiography not only to detect dilatation and aneurysms but also to diagnose aortic dissection and assess aortic valve function and cardiac function.^46^

However, conventional B-mode ultrasound has limitations in postoperative settings, especially after EVAR, due to metallic stent artefacts, limited visualization of endoleaks, and operator variability. Contrast-enhanced ultrasound (CEUS) overcomes many of these limitations (*Table 7*). It enhances delineation of the aortic lumen, aneurysm sac, and endoleak flow using microbubble contrast agents that remain strictly intravascular. Compared with CTA, CEUS has demonstrated superior sensitivity in detecting Type II endoleaks, particularly in low-flow leaks. Its use has been supported as a complementary modality in post-EVAR surveillance algorithms, notably in patients with renal insufficiency or iodinated contrast allergies.

**Table 7:** CEUS

Specificities	Details
Radiation	None
Contrast agent	Microbubbles
Field of view	Limited (not suitable for full aortic scan)
Sensitivity	Excellent for low-flow endoleaks
Best use	Post-EVAR surveillance, patients with renal failure

Recent advancements have extended CEUS applications beyond endoleak detection:

- Quantitative CEUS (using time–intensity curve analysis) allows semi-automated measurement of perfusion kinetics, potentially aiding in risk stratification of endoleaks and evaluation of sac pressurization.
- Fusion imaging combining CEUS with preoperative CTA or MRI enhances real-time anatomical guidance, particularly valuable in complex post-EVAR anatomy.
- CEUS is increasingly used to assess aortic graft patency, infection (in conjunction with inflammatory markers), and even inflammatory aortic syndromes such as aortitis, offering a bedside-friendly alternative to PET imaging.

Although CEUS remains operator-dependent and has a limited field of view compared with cross-sectional imaging, its advantages in specific patient populations and postoperative scenarios are driving wider integration into multimodality aortic imaging strategies.

## Conclusions

Modern imaging is attached to the effective management of aortic disease. From screening and diagnosis to risk stratification and postoperative surveillance, imaging guides every step of clinical decision-making. Advances in hybrid modalities and AI offer promising avenues for greater precision, automation, and personalization. As these technologies mature, they will increasingly inform tailored management strategies, enhance diagnostic accuracy, and improve patient outcomes in aortic care.

## Consent

This study is a non-systematic review of published articles for which consent was considered irrelevant by the university ethics committee because all authors of articles cited in this study had reported, where indicated, informed consent of all patients.

## Data Availability

No new data were generated or analysed in support of this research.

## Lead author biography

**Resume**

Jean-Baptiste RICCO, MD, PhD

*Born March 14, 1948*

Married †, 3 children

Professor Emeritus, University of Poitiers (2019–2024)

Member of the National Academy of Medicine

Email: jean.baptiste.ricco@univ-poitiers.fr

**Diplomas**

- Doctorate in Medicine University of Paris VII (1980)
- Fellow Northwestern University, Chicago (1983)
- PhD statistics, Paris La Sorbonne
- Full University Professor (1990)

**Fellowship (1981–1983)**

Northwestern University, Memorial Hospital, Chicago (1981–1983)

**European Diplomas**

Fellow of the European Board of Vascular Surgery (2002)

**Hospital Positions (1990–2018)**

Head of the Vascular Surgery Department, Poitiers University Hospital

**Clinical Research (2019–2025)**

President, Department of Clinical Research, Poitiers University Hospital, 2019–2021

Professor Emeritus, University of Poitiers, 2019–2026

Consultant for Clinical Research, Cardiovascular Center, Toulouse University Hospital,

2022–2026

**Scientific Publications**

ORCID identifier: 0000-0003-1912-900X

293 publications referenced in PubMed

H index: 55

Citations: 25 180

**Honours**

- Laureate of the Fulbright Foundation, USA (1981)
- Secretary general of the French Society of Vascular Surgery (1990–1996)
- President of the French Society of Vascular Surgery (1999–2000)
- Editor-in-Chief of the *European Journal of Vascular and Endovascular Surgery* (2010–2013)
- President of the European Society for Vascular Surgery (2014)
- Member of the French Academy of Medicine (2021)
- Commander of the Legion of Honor (2022)

**Current Positions**

- Professor Emeritus, University of Poitiers (2019–2027)
- Visiting professor: University of Rome, ‘La Sapienza’ (2020–2025)
- Scientific advisor, Toulouse University Hospital (2022–2027)
- Scientific advisor, Ministry of Health, Jury PHRC (since 2020)
